# Operator Radiation Exposure in Cone-Beam Computed Tomography Guidance

**DOI:** 10.5334/jbr-btr.816

**Published:** 2016-02-26

**Authors:** SJ Braak, MJL Strijen van, E Meijer, JPM Heesewijk van, WPThM Mali

**Affiliations:** 1 MRON (prev St. Antonius Hospital), NL; 2 St. Antonius Hospital, NL; 2 UMCU, NL

**Keywords:** effective dose, staff, needle intervention, cone beam CT, radiation shielding

## Abstract

**Objectives:**

Quantitative analysis of operator dose in cone-beam computed tomography guidance (CBCT-guidance) and the effect of protective shielding.

**Methods:**

Using a Rando phantom, a model was set-up to measure radiation dose for the operator hand, thyroid and gonad region. The effect of sterile radiation-absorbing drapes and ceiling/couch shielding was measured. Using this model we calculated the dose, based on relevant clinical parameters. The procedures were divided in thoracic and abdominal group. Furthermore, dosimetry measurements were performed during clinical cases to correlate with our calculations.

**Results:**

One hundred thirteen procedures were included between December 2007 and January 2010 (47 thoracic, 66 abdominal). The mean hand doses were 34.2 and 54.6 µSv (thoracic/abdominal respectively). The thyroid and gonad regions doses were 83.2 and 34.3 µSv in the thoracic, and 66.2 and 47.2 µSv in the abdominal group. Combined shielding reduced the dose by 98.2–98.9% (p<0.05). The radiation dose in clinical setting in the thoracic group (n=17) was 32.9 µSv (hand), 11.4 µSv (thyroid) and 16.0 µSv (gonad region). In the abdominal group (n=20) the doses were 43.4, 21.7 and 18.8 µSv respectively.

**Conclusion:**

The operator dose in CBCT-guidance without shielding is quite low, compared to the literature. Based on our data, between 375–830 cases can be performed staying below the yearly limit of 20 mSv effective whole-body dose.

## Introduction

Scatter radiation of fluoroscopy or fluorography is the major source of radiation dose for the interventional radiologist during procedures. There is an increase in X-ray fluoroscopy/fluorography use in the angiosuite, because of increasing complexity of procedures performed. There are many reports concerning the radiation dose for the staff in the angiosuite. Only a small part of the described performed procedures concerned percutaneous needle interventions [1,2].

For biopsies and drainages the interventional radiologist can use ultrasound, computed tomography fluoroscopy (CTF), Magnetic resonance imaging and fluoroscopy or a combination. In most cases ultrasound or CTF is used. By using ultrasound there is obviously no radiation exposure but many lesions are inaccessible by ultrasound due to the superposition of gas or bone. In these instances CTF is then commonly used. The X-ray exposure for radiologists during CTF guided interventions has been investigated by many authors. A mean dose outside the lead aprons of 71 µSv is reported [3,4].

Since the introduction of flat panel detectors in the angiosuite, the capability of performing a soft tissue cone-beam CT (CBCT) is available, combined with needle planning software and overlay imaging with fluoroscopy allowing to perform real-time guided needle interventions [5,6]. During a CBCT-guided intervention the interventional radiologist potentially stands relatively close to the X-ray beam to position the needle (that is, compared to most common vascular interventional procedures). During CBCT guidance the C-arm also takes an oblique and possibly lateral position resulting in a possible higher scatter radiation dose. There is currently no literature available on studies investigating the amount of radiation exposure for the operator during these CBCT-guidance interventions. In order to determine the radiation dose for the interventional radiologist during CBCT guidance, we performed a prospective study to quantify scatter radiation dose. This was based on a phantom study and correlated with a prospective study on a cohort of clinical patients. The effects of scatter radiation protection methods (standardly equipped couch and ceiling shielding; disposable radiation protection drapes) for the operator were separately measured.

## Materials and methods

Written informed consent was obtained from all patients and the institutional review board approved this prospectively performed study design.

### Cone-Beam CT-guidance Procedure

**Figure 1:**
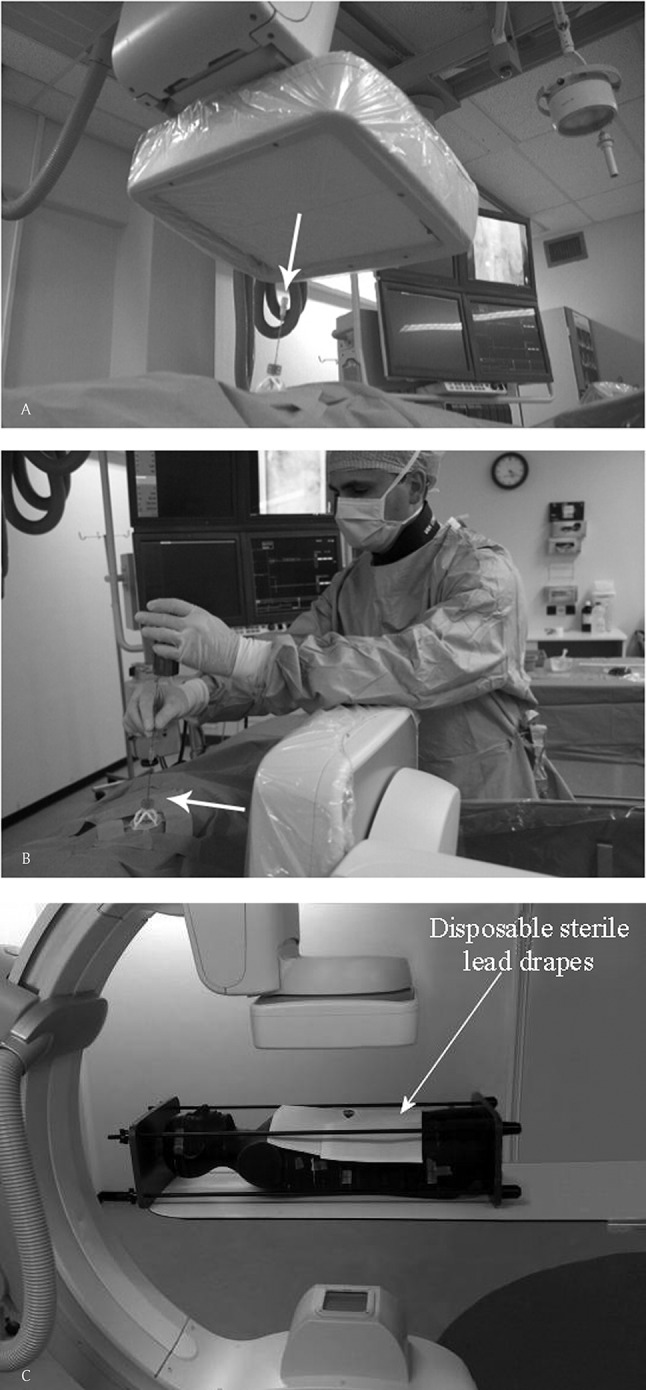
Cone-Beam CT-guidance procedure on a 59-years-old male with a suspected mass in the hilum of the right kidney. Histopathological result revealed a small cell neuroendocrine carcinoma for which this patient underwent a nephrectomy. (A) Entrypoint (EP) geometric configuration of the C-arm, looking ‘down-the-barrel’ (with arrow). (B) shows the common geometric configuration of the C-arm in the Progress view (PV). This clearly shows the close position of the operator to the radiation beam.

Cone-Beam computed tomography-guidance (CBCT-guidance) is based on a flat-panel detector C-arm angiosuite system (XperCT and XperGuide, Allura FD20). A soft tissue 3D volume is created during a 4–10 seconds rotation (240 degrees) around the patient. Within this calibrated 3D soft tissue data set the operator determines a needle access route from the skin to the target lesion, avoiding critical structures. This needle path determines the angulation and rotation of the C-arm for visualizing entry point and progression views. The fluoroscopy image is co-registered with the relevant needle path slice of the CBCT, making it possible to position and advance the needle in real time. There are two main C-arm positions; one looking right on top of the trajectory – entry-point view or EP (Figure 1a and 1b). The second view is perpendicular to the planned needle path with parallax correction – progress view or PV (Figure 1c and 1d). During the acquisition of the CBCT the operator steps outside of the angiosuite. For positioning and progressing the needle the radiologist uses fluoroscopy and has to stand close to the patient, needle and X-ray beam. After selecting the first view the needle can be placed within a safety margin of 5 mm, however the depth of the needle cannot be monitored real time in this view. After securing the adequate EP position of the needle the second position (PV) is selected to progress the needle until the planed depth is reached.

The total filtration during fluoroscopy is 1.0 mm Al and 0.9 mm Cu. The X-ray focal spot-image detector distance (FSD) is 119.5 cm. The distance from the focal spot to the center of rotation is 81 cm. The operator can choose the field diameter (FD) and the amount of collimation. The position of the interventional radiologist depends on the C-arm geometry.

### Scatter Radiation Model

**Figure 2:**
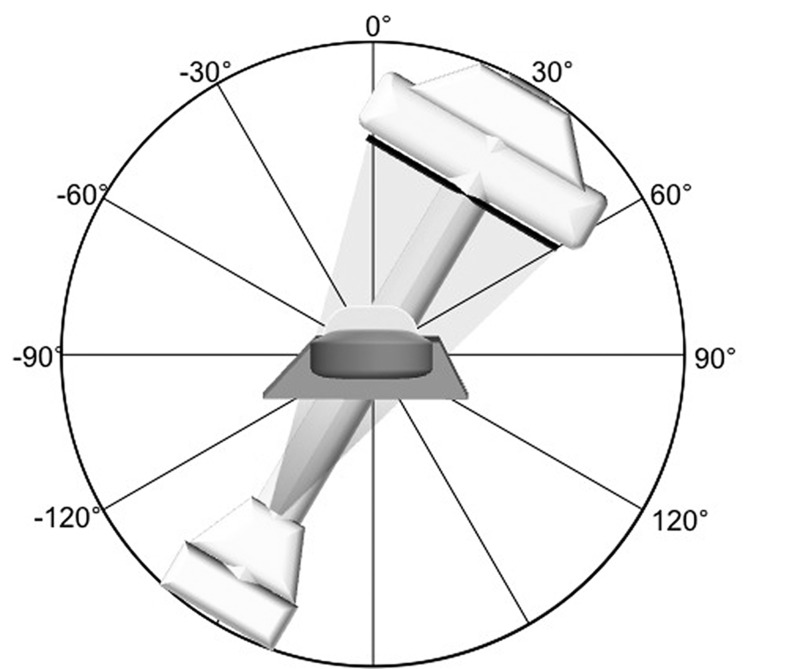
The disposable lead drape armor (Pb equivalent of 0.25 mm) as positioned during the model measurement with a Rando-phantom®.

**Figure 3:**
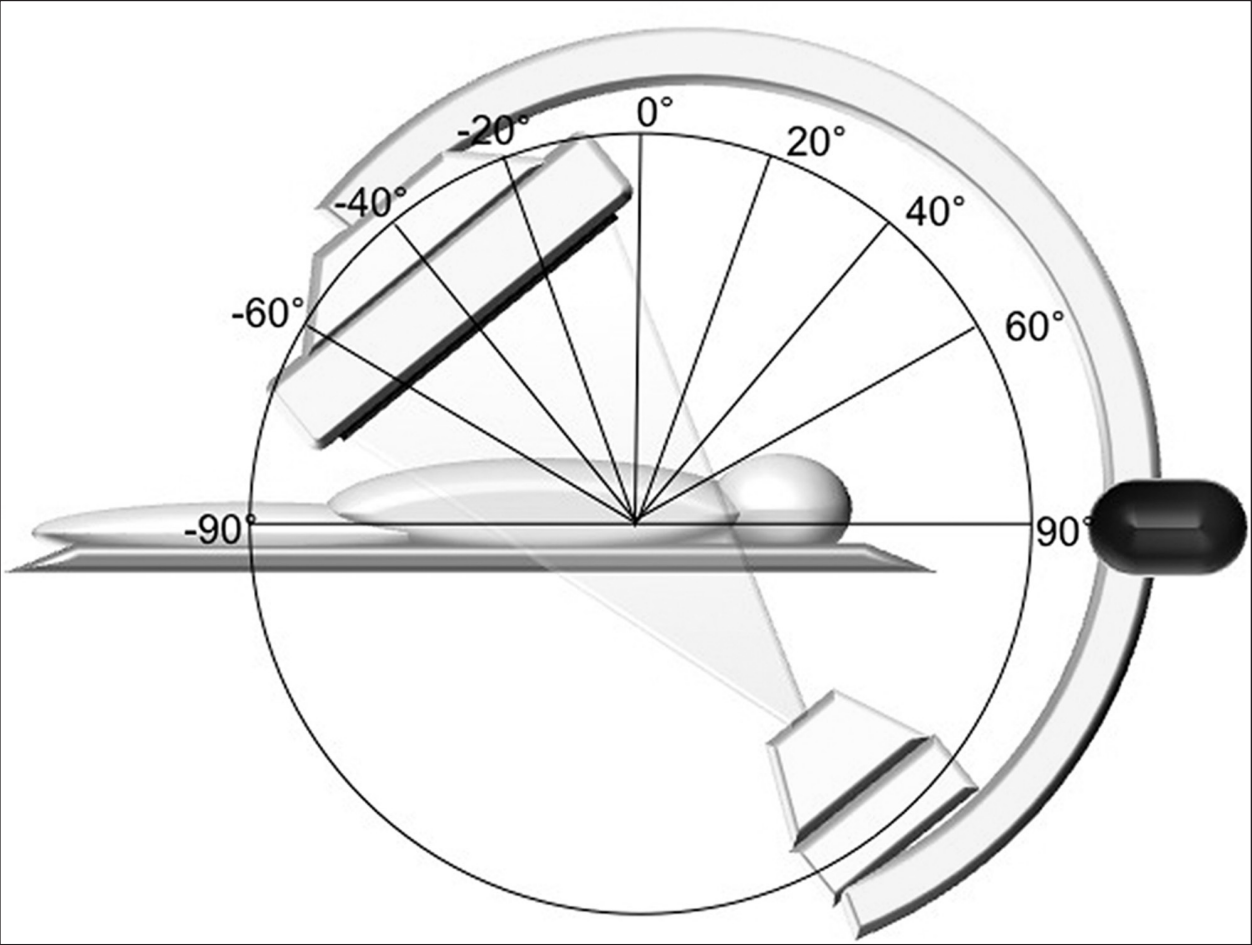
The rotation (A) and angulation (B) of the C-arm used during the model measurements of scatter radiation for the operator. Dose measurement was performed in every combination of rotation projections between -120 degrees to +120 degrees at an interval of 30 degrees and in every angulation projection with an interval of 20 degrees in a range between -40 degrees to +40 degrees.

To set up a scatter-radiation model during CBCT-guidance we used a human-shaped phantom (Rando phantom). This phantom represents a 175 cm tall and 73.5 kg (body mass index: 24 kg.m–2) hermaphrodite. We measured the scatter radiation dose of the interventional radiologist to the thyroid region (150 cm above ground), gonad region (90 cm above ground) and hand region (next to the needle, close to the direct X-ray beam) of the operator by using electronic personal dosimeters or EPD (Thermo Scientific Mk2). This dosimeter measures the penetrating dose for the thyroid and gonad region (Hp(10), µSv), i.e. the individual dose at a depth of 10 mm tissue and the skin dose for the hand region (Hp(0.07), µSv), i.e. the dose at a depth of 0.07 mm tissue. Cumulative dose value is displayed on the LCD display of the EPD [3]. The distance of the operator to the radiation source depends on the angulation and rotation of the C-arm. For every geometric position of the C-arm, a clinically representative position was chosen to measure the operator dose based on the position during equal clinical procedures (50 cm from center point of C-arm outside of the rotational arc), and which is dependent upon the geometric position of the C-arm near the tube of flat-panel detector. These positions were simulated static point with an EPD measurement device fixed to an IV pole. No human volunteer was present during the phantom study at the measurements points. We also measured the scatter dose behind ceiling- and couch-attached shielding (0.5 mm Pb equivalent), as well as using sterile disposable radiation protection drapes (Microtek Medical Drape Armour; 0.25 mm Pb equivalent, 41x25 cm) which are placed on top of the patient around the needle (Figure 2) and a combination of radiation protection drapes and shielding. We measured the dose in every combination of rotation projections of the detector between -120 degrees to +120 degrees at an interval of 30 degrees, to simplify the measurements. In every angulation projection the dose was measured with an interval of 20 degrees in a range of -40 degrees to +40 degrees (Figure 3). In every geometric setting the scatter radiation was measured during 15 seconds of fluoroscopy. After every 15 seconds of fluoroscopy the EPD’s display was read and recorded. The effect of changing the FD and collimation (0% or 50% collimation) in every combination of geometry and shielding was also determined. The above measurements were performed in the abdominal region and thoracic region of the phantom. This resulted in a scatter dose rate (µSv/s) for every combination of geometric setting, FD, collimation, ceiling/couch shielding and sterile disposable radiation protection drapes.

### Patients

Written informed consent was obtained from all patients and the institutional review board approved the study design. Patients were included from December 2007 and January 2010. All patients (N=186) included in this study had an indication for a CT-guided needle intervention (lesions not accessible by ultrasound due to overlaying gastro-intestinal gas, bony structures or lesions in the lung parenchyma) and underwent a percutaneous needle intervention using CBCT guidance. There was no target size limit. Because the phantom used in our model had a body mass index (BMI) of 24 kg.m^–2^, we selected only patients with BMI ranging from 22–26 kg.m^–2^. In total 113 patients were included. The baseline data of this population is shown in Table 1. The CBCT-guidance procedures were performed by two radiologists (SB & MvS) with five and eleven years experience and equal experience in CBCT-guidance. The procedures were divided into two groups: thoracic (n=47) and abdominal procedures (n=66). During the procedures, relevant parameters were prospectively recorded (e.g. C-arm geometry, fluoroscopy time, FD and collimation).

**Table 1:** Overview of the characteristics used for the model and during the clinical cases.

		Thorax	Abdomen	total

Scatter radiation model	N	47	66	113
	Sex	19 women / 19 men	28 women / 47 men	47 women/ 66 abdomen
	Mean age (y)	62.1 (range 24–85)	63.9 (range 32–83)	63.3 (range 24–85)
	Mean target size (mm)	31.0 (95% CI 24.3–37.6)	25.1 (95% CI 20.1–30.1)	27.8 (95% CI 23.8–31.8)
	Mean BMI (kg/m^2^)	23.9 (95% CI 23.5–24.3)	23.9 (95% CI 23.5–24.3)	23.9 (95% CI 23.8–24.0)
Clinical cases	N	17	20	37
	Sex	5 women / 12 men	8 women / 12 men	13 women / 24 men
	Mean age (y)	67.3 (range 35–83)	62.6 (range 35–83)	64.7 (range 35–83)
	Mean length size (mm)	23.7 (95% CI 19.8–30.1)	22.9 (95% CI 17.7–27.5)	23.3 (95% CI 20.5–27.2)
	Mean BMI (kg/m^2^)	24.4 (95% CI 23.4–25.5)	24.0 (95% CI 22.9–25.0)	24.2 (95% CI 23.5–24.9)

### Scatter Radiation Dose Calculation

Using the scatter radiation model, we extrapolated the dose for the radiologist at the thyroid region, gonad region and hand region during the performed CBCT-guidance procedures performed in the patient population. The scatter radiation dose for the operator depends on the geometric position of the C-arm, irradiated target volume, parameters of the system (e.g. kV, mAs, fluoroscopy pulse rate, filtering, collimation, FD and fluoroscopy time), operator position and used radiation protection. During the performed CBCT-guidance procedures we registered the necessary data. In the scatter radiation model all the scatter radiation dose dependent parameters between the model and patients (e.g.. C-arm geometry, irradiated volume, system parameters, operator position, radiation protection) were matched except the actual fluoroscopy time. For this we used the determined scatter radiation dose rate (µSv/s) at the designated and correlated measurement points. Based on the details obtained during the CBCT-guidance procedures and the scatter radiation model we could extrapolate the scatter dose by multiplying the fluoroscopy time (sec) of the detailed obtained data during CBCT-guidance procedures with the scatter radiation dose rate (µSv/s) measured in the model, resulting in a scatter radiation dose in µSv per procedure.

### Real-time Dose Measurement

In June 2010 a Dose Aware System or DAS (Philips Healthcare) was introduced in our radiological department. In the period between June and November 2010 we performed 37 CBCT-guidance procedures in which we used this system. Baseline data of these patients are shown in Table 1. The dosimeter measures the scatter dose rate (µSv/h) and cumulative scatter dose (µSv) and sends this information every second wirelessly to a base station. The dosimetry data from the base station was downloaded to a laptop for further analysis. The dosimeters were worn by the interventional radiologist at the same position as the position of the dosimeters during the scatter radiation phantom measurement (thyroid region (150 cm), gonad region (90 cm) and hand (under a sterile glove at the dorsum of the hand) that placed and progressed the needle (closest to the beam). The position of the operator was at the same position where we measured the scatter radiation in the model, which was near the tube of the flat-panel detector. During these clinical procedures we routinely worked with ceiling- and couch-mounted protective lead shielding.

### Analyses

Microsoft Office Excel 2007 was used for registering and summarizing the results, and SPSS software was used for the statistical analysis. The magnitudes of the differences between the mean of the different groups of shielding were analyzed with a paired-sample T-test. The differences were considered significant for *p* values less than 0.05.

## Results

The mean fluoroscopy time, kV, mAs, FD, angulation and rotation for the EP position, PV position in the thorax and abdomen group are summarized in Table 2.

**Table 2:** Overview of the data in entrypoint view, progress view and in total. Data is based on the clinical setting and used for the scatter radiation model.

	Entrypoint		Progress View		Total	
	
	Thorax	Abdomen	Thorax	Abdomen	Thorax	Abdomen

Fluoroscopy time (s); median (95% CI)	74.4 (64.6–84.3)	68.3 (62.4–74.1)	137.9 (124.8–150.9)	111.4 (103.9–118.8)	212.3 (192.7–231.9)	179.7 (168.7–190.6)
kV; median (95% CI)	91.9 (90.4–93.3)	98.6 (97.2–100.1)	103.3 (101.2–105.3)	117.7 (117.0–118.4)		
mAs; median (95% CI)	13.5 (13.3–13.7)	14.7 (14.5–14.8)	14.9 (14.6–15.1)	15.4 (15.3–15.6)		
Field Diameter (cm) ; median (95% CI)	27.7 (25.3–30.1)	28.0 (26.0–30.0)	27.7 (25.6–29.7)	31.1 (29.5–32.8)		
Angulation; median (95% CI)	–3° (–8°–+2°)	11° (7°–15.0°)	0° (–0°–0°)	0° (0°–0°)		
Rotation; median (95% CI)	–4° (–12°–+5°)	–4.0° (–10°–+2°)	–22° (–44°–1°)	–25° (–45°– –6°)		
Dose Hand (µSv); median (95% CI)	10.7 (8.2–13.1)	17.9 (13.2–22.6)	23.5 (17.4–29.7)	36.7 (30.9–42.6)	34.2 (26.9–41.5)	54.6 (46.9–62.4)
Dose Thyroid region (µSv); median (95% CI)	68.6 (–3.4–140.5)	42.6 (9.1–76.1)	13.8 (9.2–18.4)	23.6 (17.7–29.5)	83.2 (11.1–155.3)	66.2 (31.4–100.9)
Dose Gonads region (µSv); median (95% CI)	17.8 (11.1–24.4)	20.8 (17.2–24.4)	16.1 (11.6–20.7)	26.4 (20.9–32.0)	34.3 (26.0–42.7)	47.2 (39.9–54.5)
Dose Thyroid Shielding (µSv); median (95% CI)	1.7 (–0.1–3.4)	0.7 (0.2–1.2)	0.5 (0.3–0.6)	1.0 (0.7–1.3)	2.2 (0.4–3.9)	1.7 (1.1–2.3)
Dose Gonads Shielding (µSv); median (95% CI)	1.3 (0.9–1.7)	1.5 (1.2–1.8)	1.3 (0.9–1.7)	2.1 (1.6–2.6)	2.6 (2.0–3.2)	3.6 (2.9–4.2)
Dose Hand Drape (µSv); median (95% CI)	3.3 (2.4–4.2)	7.1 (4.1–10.0)	4.6 (3.5––5.8)	10.5 (6.3–14.8)	7.9 (6.1–9.8)	17.6 (11.9–23.4)
Dose Thyroid Drape (µSv); median (95% CI)	94.7 (–22.1–211.6)	76.8 (14.4–139.3)	16.7 (8.7–24.7)	21.8 (18.3–25.3)	111.4 (–5.9–228.7)	98.6 (35–161.4)
Dose Gonads Drape (µSv); median (95% CI)	33.8 (19.2–48.5)	34.3 (26.9–41.8)	26.4 (9.3–33.5)	37.8 (29.6–46.0)	60.2 (43.9–76.5)	72.1 (59.2–85.0)
Dose Thyroid Both (µSv); median (95% CI)	0.06 (0.00–0.14)	0.05 (0.03–0.08)	0.02 (0.01–0.02)	0.13 (0.05–0.21)	0.08 (0.01–0.16)	0.18 (0.08–0.28)
Dose Gonads Both (µSv); median (95% CI)	0.06 (0.00–0.13)	0.05 (0.02–0.08)	0.18 (0.12–0.25)	0.40 (0.24–0.56)	0.37 (0.28–0.46)	0.84 (0.55–1.13)

### Scatter Radiation Dose Calculation: Thorax Group

Radiation dose rates without radiation protection of the operator’s hand, thyroid and gonad region ranged from 0.14 to 0.18 µSv/sec, 0.06 to 0.67 µSv/sec and 0.13 to 0.18 µSv/sec respectively. The median calculated dose per procedure for the hand, thyroid region and gonad region respectively without the use of ceiling- and couch-mounted protective shielding or radiation protection drape coverage are displayed in Table 2.

The use of ceiling and couch shielding resulted in a significant dose reduction, compared to the dose without any shielding. No difference on the hand dose due to the use of ceiling and couch shielding was found. Using only the radiation protection drape reduced the hand dose significantly but increased the dose to the thyroid and gonad region. The highest reduction was achieved by using both the ceiling and couch shielding and the radiation protection drapes. In this setting the hand dose did not change compared to the use of the lead only. Detailed information of the dose differences is shown in Table 3.

**Table 3:** Thorax group.

		Couch/Ceiling Shielding	Lead drape Shielding	Couch/Ceiling and Lead drape Shielding

Hand	Entrypoint	No difference	–68.9%	No difference compared to lead drape shielding
	Progress View		–80.4%	
	Total		–76.8%	
Thyroid region	Entrypoint	–97.5%	+38.4%	–99.8%
	Progress View	–96.6%	+13.8%	–99.5%
	Total	–97.4%	+33.9%*	–99.7%
Gonad region	Entrypoint	–93.2%	+90.3%	–97.9%
	Progress View	–91.6%	+59.3%	–98.4%
	Total	–92.4%	+75.3%	–98.2%

Percentages of dose difference compared to the setting without any radiation shielding in the scatter radiation model. All percentages represent significant (p<0.05) changes expect the one marked with an asterix (*; p=0.31).

### Scatter Radiation Dose Calculation: Abdominal Group

In this group the radiation dose rates of the operator’s hand, thyroid region and gonad region ranged from 0.28 to 0.33 µSv/sec; 0.19 to 0.53 µSv/sec and 0.24 to 0.29 µSv/sec respectively. The median calculated dose per procedure for respectively the hand, thyroid region and gonad region without the use of ceiling and couch shielding or radiation protection drape coverage are displayed in Table 2.

Table 4 shows the detailed information of the dose differences. The use of ceiling and couch shielding resulted in a significant dose reduction, compared to the dose without any shielding. No difference was found on the hand dose due to the use of ceiling and couch shielding. Using only the radiation protection drape reduced the hand dose significantly, but increased the total dose to the thyroid and gonad region significantly (p<0.05). Similarly to the thoracic group in the abdominal group the highest reduction was achieved by using both the ceiling and couch shielding and the radiation protection drapes. The use of the ceiling and couch shielding had no effect on the hand dose, because the hand is before these shielding.

**Table 4:** Abdominal group.

		Couch/Ceiling Shielding	Lead drape Shielding	Couch/Ceiling and Lead drape Shielding

Hand	Entrypoint	No difference	–60.4%	No difference compared to lead drape shielding
	Progress View		–71.3%	
	Total		–67.8%	
Thyroid region	Entrypoint	–98.4%	+80.5%	–99.9%
	Progress View	–95.8%	–7.7%*	–99.8%
	Total	–97.4%	+49.0%	–99.8%
Gonad region	Entrypoint	–92.8%	+65.2%	–98.9%
	Progress View	–92.0%	+42.9%	–98.7%
	Total	–92.4%	+52.8%	–98.9%

Percentages of dose difference compared to the setting without any radiation shielding in the scatter radiation model. All percentages represent significant (p<0.05) changes expect the one marked with an asterix (*; p=0.43).

### Real-Time Dose Measurement

The median total scatter radiation dose during 17 thoracic procedures was 32.9 µSv (95% CI 16.5–49.4 µSv) for the hand, 11.4 µSv (95% CI 6.5–16.3 µSv) for the thyroid and 16.0 µSv (95% CI 6.7–25.4 µSv) for the gonad region. For the abdominal procedures (N=20) the median scatter radiation dose was 43.4 µSv (95% CI 30.3–56.5 µSv), 21.7 µSv (95% CI 11.8–31.7 µSv), 18.8 µSv (95% CI 7.1–30.5 µSv) for the hand, thyroid and gonad region respectively. Compared to the mean dose results of the scatter radiation model, the dose measured during the live cases were 11.9%, 417.5% and 132.8% lower respectively for the hand, thyroid and gonad region compared to calculated dose without any form of radiation protection. Compared to the mean dose with couch and ceiling protection of the scatter radiation model, the live doses were 86.2 and 82.4% higher for the thyroid and gonad region without any difference in the hand dose.

## Discussion

In our study, the mean radiation dose ranges from 34.2 to 83.2 µSv per procedure and radiation dose rates ranges from 0.06 to 0.67 µSv/sec; all without any radiation protection. By using radiation protection the scatter dose can be changed between +75.3% and –99,8% depending on the type of shielding (couch and ceiling shielding, lead drape or a combination) and the region of measurement (thyroid, hand or gonad). The outcome of the model provides relevant information of the scatter radiation dose without any shielding till a situation with optimal shielding protection. Overall this study provides information on the radiation dose for the radiologist using CBCT guidance and demonstrates that additional adequate lead protection can reduce the dose significantly.

The recently developed interventional tool using CBCT guidance has proven to be accurate and uses a lower radiation dose for the patient compared to CT guidance. However, there is to our knowledge currently no data on the scatter radiation dose for the interventional radiologist in CBCT guidance [6,7,8,9,10]. Many studies are performed on scatter radiation dose during interventional procedures, but these concern fluoroscopic interventions or CT-fluoroscopy guided interventions.

A recent study by Martin et al. [1] reviewed the staff dose over the last 20 years. He concluded that there is a wide range of radiation dose to the hand and thyroid reported in the literature. The reported mean scatter dose in his review is 350 µSv per procedure for the hand and approximately 97.5 µSv for the thyroid. Percutaneous procedures result in the highest dose, however these procedures were not the majority of the interventions. During procedures with percutaneous access, the reported mean hand dose is 920 µSv. These procedures were mainly transjugular intrahepatic portosystemic shunts (TIPS), biliary drainages and nephrostomy procedures. These high operator radiation doses are the result of long fluoroscopy times but not due to the radiation beam geometry [11,12,13]. In CBCT-guidance the fluoroscopy time short, resulting in a lower radiation dose to the interventional radiologist. However the wide range of the C-arm geometry can result in high scatter radiation during CBCT-guidance. This is the same in coronary interventional procedures where the radiation beam projections have a wide range in C-arm geometry with high scatter radiation for the cardiologist, especially in the beam projection where the tube is nearest to the operator [2,14]. Depending on the beam projection, the maximum radiation dose rate is reported between 0.83–3.3 µSv/sec for the head, waist and knee region [14], which is higher than our reported personal dose and dose rate. The reason for this is probably the geometric C-arm position during coronary procedures (tube near the operator).

Needle guidance using conventional CT can be performed without any radiation dose to the operator and is considered to be accurate and safe, but is however time consuming [15]. With the introduction of CT fluoroscopy, first described in 1994 by Katada et al. [16], real-time visualization was possible making needle placement quicker, at least with the same accuracy, but resulting in a higher radiation dose to the radiologist [15]. There is a large variation in the reported scatter radiation dose during CT-fluoroscopy guidance procedures, due to different CT-fluoroscopy settings, use of needle guidance devices, radiation protection drapes, operator experience and method and region of measurement [4]. The reported dose per CT-fluoroscopy guided procedure is between 7–2200 µSv and dose rate between 0.2–39.5 µSv/sec [3,4]. Using CBCT guidance the highest dose rate is in progress view with the tube near to the interventional radiologist. This dose rate is 0.67 µSv/sec, which is well in the lower part of the reported CT-fluoroscopy dose rate. In this position the gonad region is behind the tube, which in itself absorbs the dose but the thyroid region is in the peak scatter region.

Compared to the live cases we found a considerable difference in the dose, especially for the thyroid and gonad region. A probable explanation for this is that the calculated doses are, despite being clinical representative measurement points, in fact static points. During a live case the operator will move and the use of shielding is not always maximal. Therefore the measurement in the scatter radiation model with shielding is definitely lower because optimal shielding was always present. However, the extrapolated dose, without any form of shielding, still results in a very low scatter radiation dose for the operator. To reach the maximal annual dose limit (of 20 mSv whole body effective dose in Europe) an interventional radiologist can perform approximately 240 cases a year [17,18,21,22]. Considering this, the model without shielding overestimated the dose and the true scatter radiation dose will be in the lower half between the dose without shielding and with shielding. Despite the quite low doses in the model without the shielding, the use of shielding is always recommended.

Using radiation shielding (couch and ceiling) the scatter dose can be reduced by 25–99% [2,14,19,20]. In our study we found dose reductions comparable to the reported upper limit in the literature. Disposable radiation protection drapes are reported to reduce the scatter dose, but this was during CT-fluoroscopy guided procedures [21,22]. Using only disposable radiation protection drapes we found that the total scatter dose is even higher for the thyroid and gonad region than without any shielding. Probably this is because the drapes are only horizontally on top of the patient and result in more scatter because of the automatic fluoroscopy settings of the system. Using both the disposable radiation protection drapes and the couch and ceiling shielding the additional effect of the radiation protection drapes is significant. Also the dose to the patient can be altered due to these disposable radiation protection drapes because of alteration of the exposure factors, but this should be further investigated.

It should be emphasized that the scatter radiation dose to the radiologist reported in this study represents the dose outside a protective lead gown. Because of the ALARA (as low as reasonably achievable) principle, the interventional radiologists wore appropriate lead aprons and thyroid shielding despite the fact that scatter dose during CBCT-guidance procedures was low. Because of these protective measurements the dose will be further reduced. Lead gloves for the hands are available, but they are not commonly used during the procedures because this decreases the touch and feel of the operator, which is generally considered undesirable.

A possible limitation to this study is the validity of the assumption that the phantom is a real patient. This type of phantom is however widely used to measure patient dose and operator dose; therefore we believe the results of this study are representative and in line with published data [14,19]. Also one can comment that for comparison of the extrapolated radiation dose we used a relatively small live cases group. Our reported scatter radiation dose is also only suitable for patient with a BMI of 22–26 kg.m^–2^. Nowadays multiple vendors offer commercially available CBCT-guidance solutions. Our finding concerns only one vendor. Comparison with other vendor systems should be performed to determine the difference in dose between the other systems.

Furthermore experienced CBCT-guidance users produced the data for the scatter dose calculation. In first time users with a learning curve in using CBCT guidance the dose will probably be higher in the beginning.

We expect that the scatter radiation dose can be even lower in the future by adjusting the fluoroscopy settings (e.g. kV, mAs and lowering the pulse rate) [19]. Also asymmetrical collimation could contribute but is currently not available in this system. When performing a procedure with the needle trajectory at the edge of the imaging field little to no collimation can be used.

## Conclusion

We determined the radiation dose for the interventional radiologist based on a scatter radiation model and clinical cases. The mean scatter radiation dose without shielding in the scatter model was 53.3 µSv, which is quite low compared to the reported scatter radiation dose. Compared to the clinical cases (mean dose 24.0 µSv), the scatter model without shielding overestimated the radiation dose. Based on the model and real-time measured clinical cases between 375–830 cases a year can be performed staying below the mean yearly limit of 20 mSv effective dose (whole body) in Europe. The best significant dose reduction of 98.6% can be achieved by a combination of radiation protection drape and ceiling and couch shielding.

## Competing Interests

The authors declare that they have no competing interests.

## References

[1] Martin CJ (2009). A Review of Radiology Staff Doses and Dose Monitoring Requirements. Radiat Prot Dosimetry.

[2] Häusler U, Czarwinski R, Brix G (2009). Radiation exposure of medical staff from interventional X-ray procedures: a multicentre study. Eur Radiol.

[3] Teeuwisse WM, Geleijns J, Broerse JJ, Obermann WR, van Persijn, van Meerten EL (2001). Patient and staff dose during CT guided biopsy, drainage and coagulation. Br J Radiol.

[4] Buls N, Pagés J, de Mey J, Osteaux M (2003). Evaluation of patient and staff doses during various CT Fluoroscopy guided interventions. Health Phys.

[5] Racadio JM, Babic D, Homan R, Rampton JW, Patel MN, Racadio JM (2007). Live 3D guidance in the interventional radiology suite. AJR Am J Roentgenol.

[6] Braak SJ, van Strijen MJ, van Leersum M, van Es HW, van Heesewijk JP (2010). Real-Time 3D fluoroscopy guidance during needle interventions: technique, accuracy, and feasibility. AJR Am J Roentgenol.

[7] Lee WJ, Chong S, Seo JS, Shim HJ (2012). Transthoracic fine-needle aspiration biopsy of the lungs using a C-arm cone-beam CT system-diagnostic accuracy and post-procedural complications. Br J Radiol.

[8] Hwang HS, Chung MJ, Lee JW, Shin SW, Lee KS (2010). C-arm cone-beam CT-guidance percutaneous transthoracic lung biopsy Usefulness in evaluation of small pulmonary nodules. AJR Am J Roentgenol.

[9] Braak SJ, van Strijen MJ, van Es HW, Nievelstein RA, van Heesewijk JP (2011). Effective dose during needle interventions: cone-beam CT guidance compared with conventional CT guidance. J Vasc Interv Radiol.

[10] Hirota S, Nakao N, Yamamoto S, Kaboyashi K, Maeda H, Ishikura R (2006). Cone-Beam CT with Flat-Panel-Detector Digital Angiographic system early experience in abdominal interventional procedures. Cardiovasc Intervent Radiol.

[11] Williams JR (1997). The interdependence of staff and patient doses in interventional radiology. Br J Radiol.

[12] Ginjaume M, Pérez S, Ortega X (2007). Improvements in extremity dose assessment for ionising radiation medical applications. Radiat Prot Dosimetry.

[13] Whitby M, Martin CJ (2005). A study of the distrubution of dose across the hands of interventional radiologists and cardiologists. Br J Radiol.

[14] Morrish OWE, Goldstone KE (2008). An investigation into patient and staff doses from X-ray. Br J Radiol.

[15] Silverman SG, Tuncali K, Adams DF, Nawfel RD, Zou KH, Judy PF (1999). CT Fluoroscopy-guided Abdominal Interventions Techniques, Results, and Radiation Exposure. Radiology.

[16] Katada K, Anno H, Takeshita G, Ogura Y, Koga S, Ida Y (1994). Development of Real-time CT Fluoroscopy. Nippon Acta Radiologica.

[17] Wrixon AD (2008). New ICRP recommendations. J Radiol Prot.

[18] ICRP (2007). Recommendations of the International Commission on Radiological Protection. ICRP Publication 103. Ann. ICRP.

[19] Kuon E, Schmitt M, Dahm JB (2002). Significant reduction of radiation exposure to operator and staff during cardiac interventions by analysis of radiation leakage and improved lead shielding. Am J Cardiol.

[20] Marx MV, Nikiason L, Mauger EA (1992). Occupational radiation exposure to interventional radiologists a prospective study. J Vasc Interv Radiol.

[21] Stoeckelhuber BM, Leibecke T, Schulz E, Melchert UH, Bergmann-Koester CU, Helmberger T (2005). Radiation Dose to the Radiologist’s Hand During Continuous CT Fluoroscopy-Guided Interventions. Cardiovasc Intervent Radiol.

[22] Nawfel RD, Judy PF, Silverman SG, Hooton S, Tuncali K, Adams DF (2000). Patient and Personnel Exposure during CT-fluoroscopy guided interventional procedures. Radiology.

